# Supplementary material for: Trends in developmental milestone attainment among Israeli children between 2019 and 2024
Source: Isr J Health Policy Res. 2026 Jul 16;15:28. doi: 10.1186/s13584-026-00771-2 (PMC13374192; doi:10.1186/s13584-026-00771-2)
Supplement: Supplementary file 2 — Supplementary Material 2 [file 13584_2026_771_MOESM2_ESM.docx]

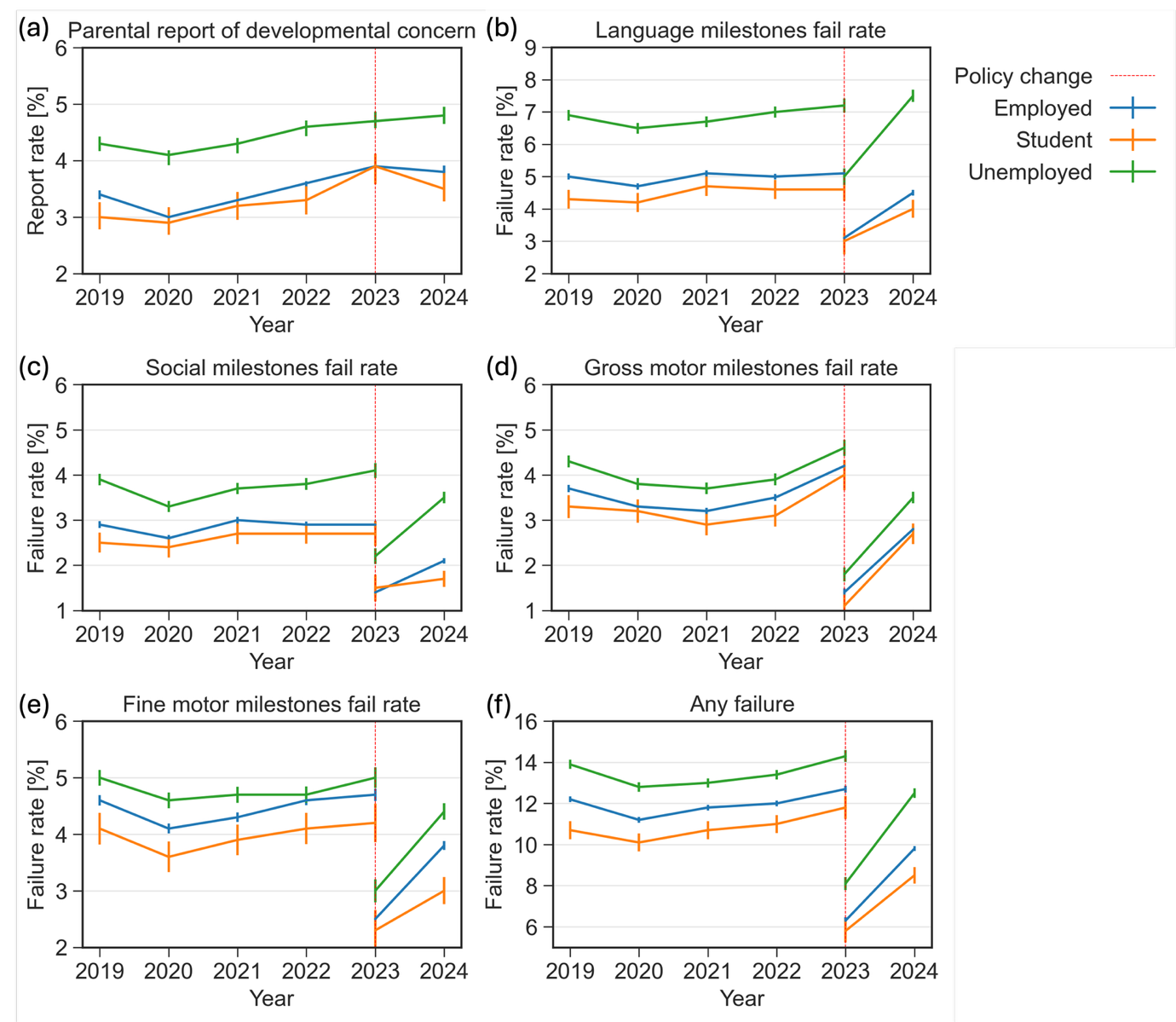


Supplementary Figure 1. Reports of concern and failure rates in various developmental domains stratified by maternal employment status between 2019-2024. (a) Parental report of developmental concern (b) Failure rate in the language domain (c) Failure rate in the social domain (d) Failure rate in the gross motor domain (e) Failure rate in the fine motor domain (f) Failure rate in any developmental domain


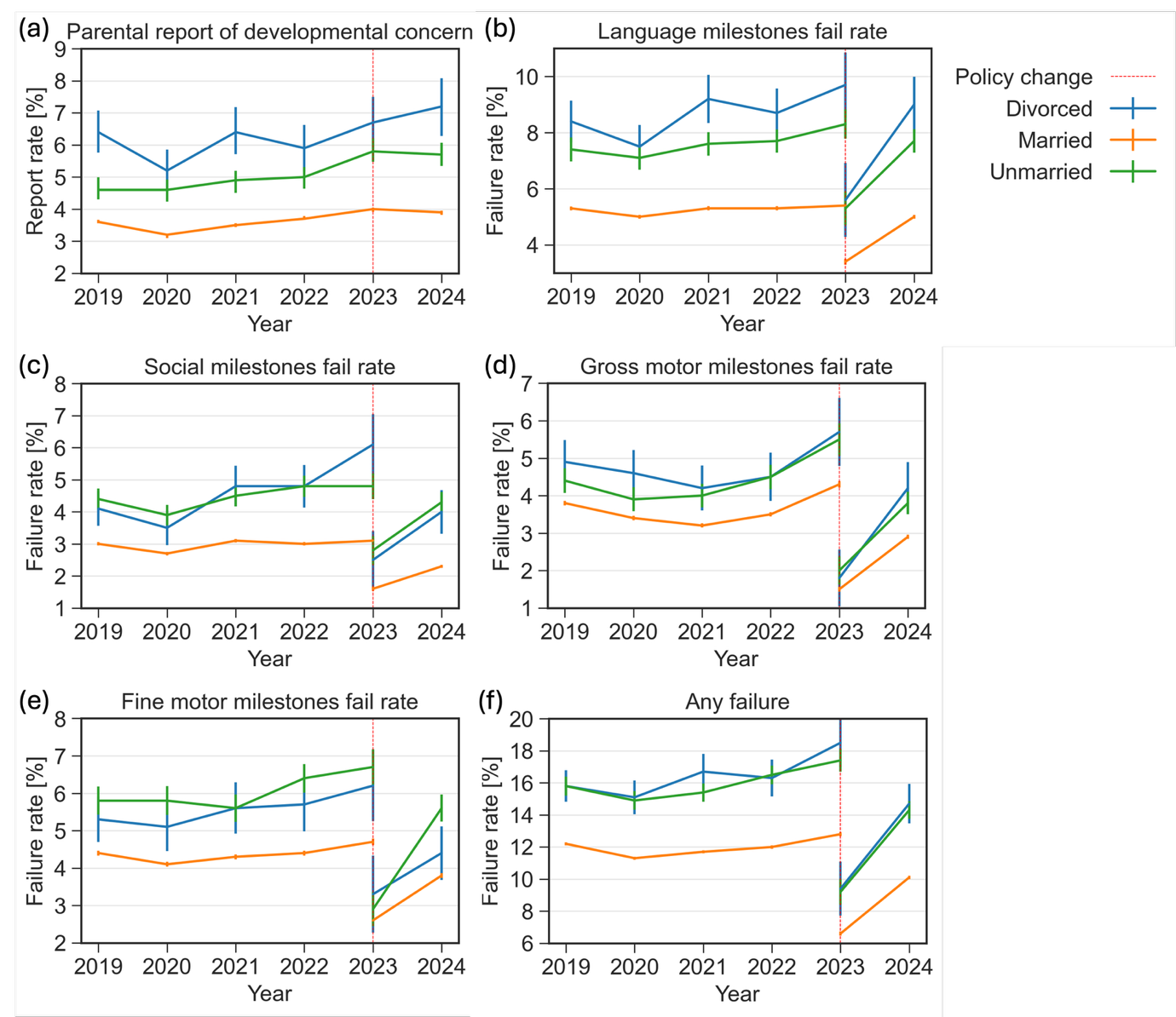


Supplementary Figure 2. Reports of concern and failure rates in various developmental domains stratified by maternal marital status between 2019-2024. (a) Parental report of developmental concern (b) Failure rate in the language domain (c) Failure rate in the social domain (d) Failure rate in the gross motor domain (e) Failure rate in the fine motor domain (f) Failure rate in any developmental domain


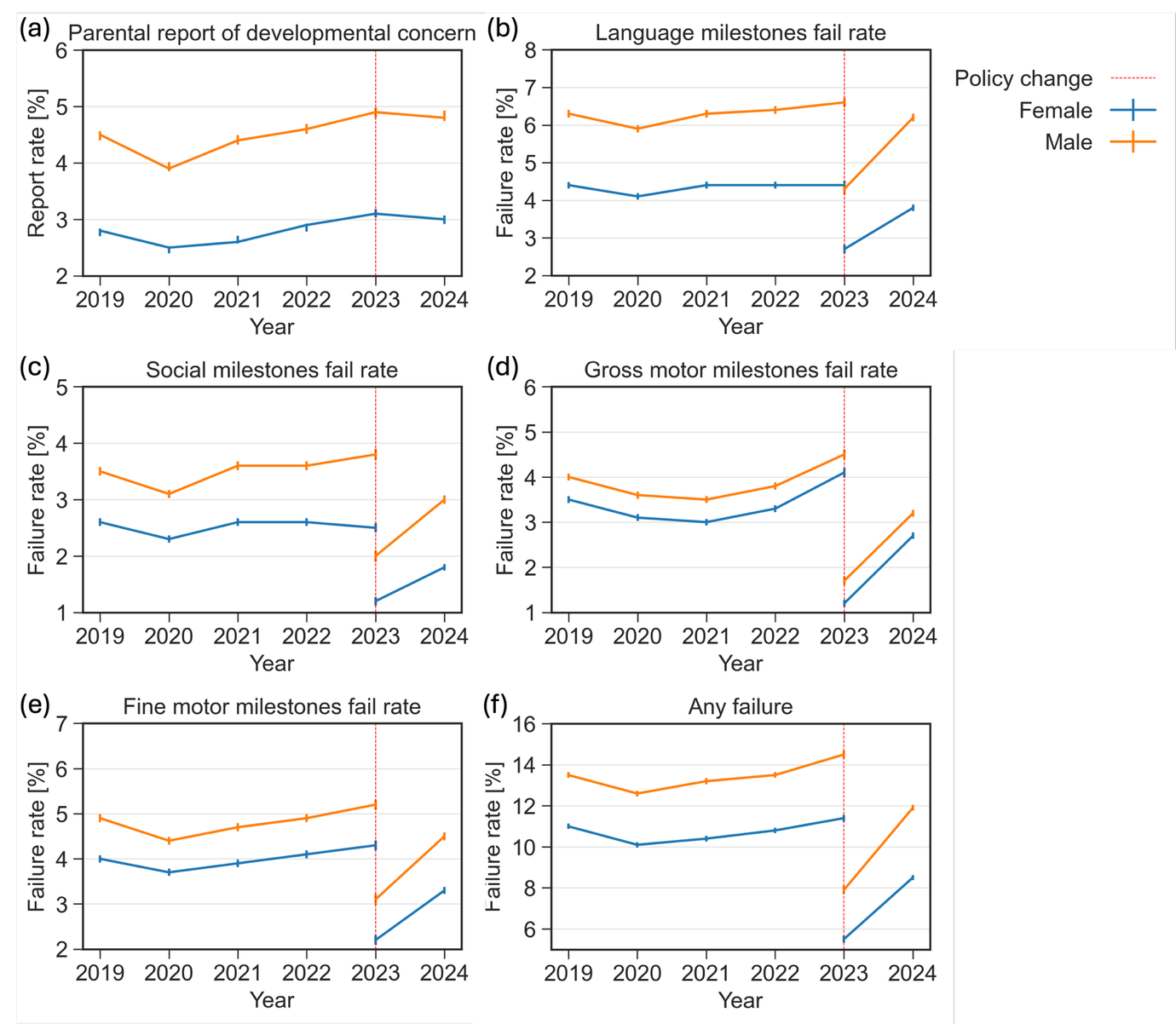


Supplementary Figure 3. Reports of concern and failure rates in various developmental domains stratified by child’s sex between 2019-2024. (a) Parental report of developmental concern (b) Failure rate in the language domain (c) Failure rate in the social domain (d) Failure rate in the gross motor domain (e) Failure rate in the fine motor domain (f) Failure rate in any developmental domain


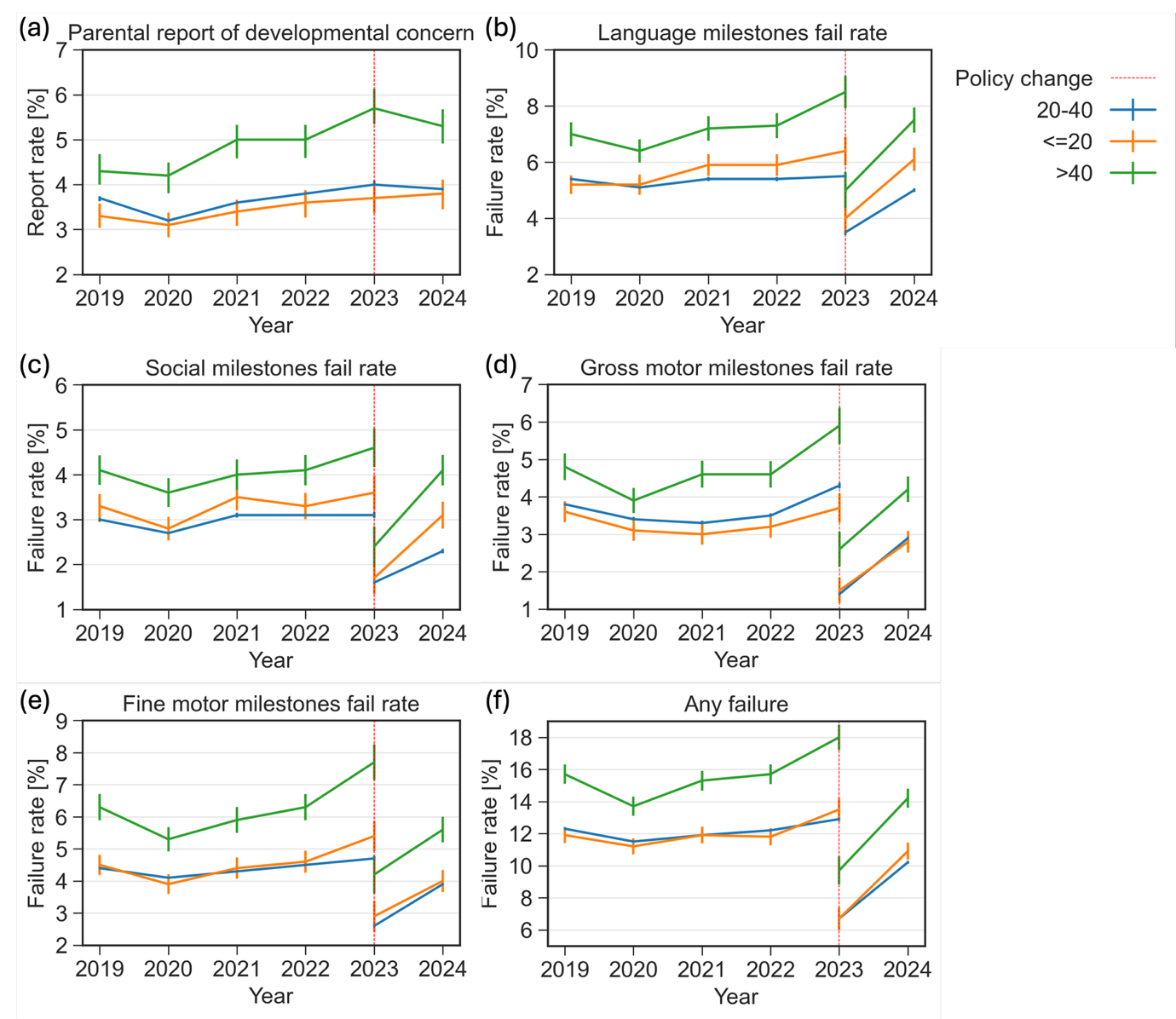


Supplementary Figure 4. Reports of concern and failure rates in various developmental domains stratified by maternal age at childbirth between 2019-2024. (a) Parental report of developmental concern (b) Failure rate in the language domain (c) Failure rate in the social domain (d) Failure rate in the gross motor domain (e) Failure rate in the fine motor domain (f) Failure rate in any developmental domain


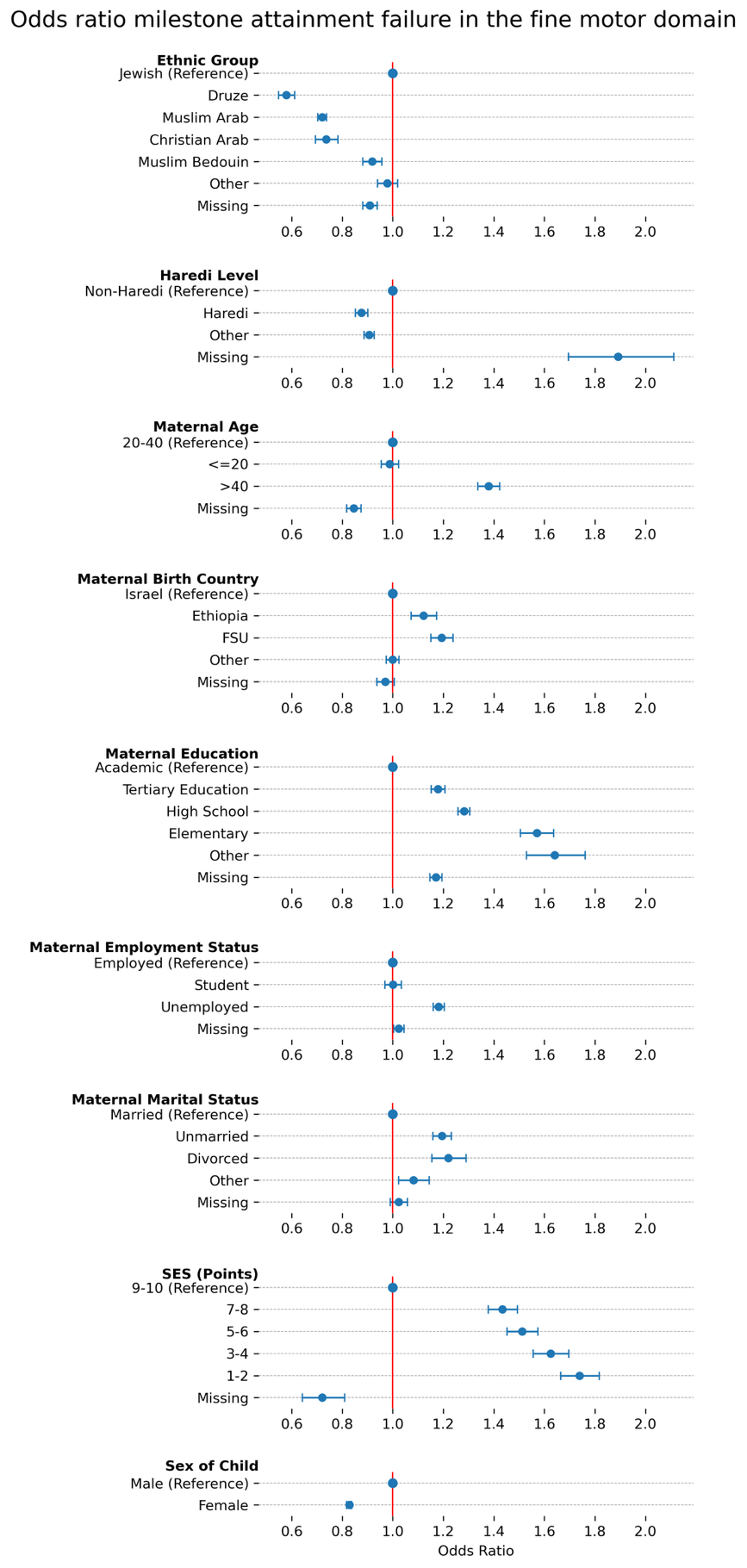


Supplementary Figure 5. Multivariable analysis for failure in the fine motor domain for 2019-2022, where x axis is the odds ratio and y axis is the demographic variables.


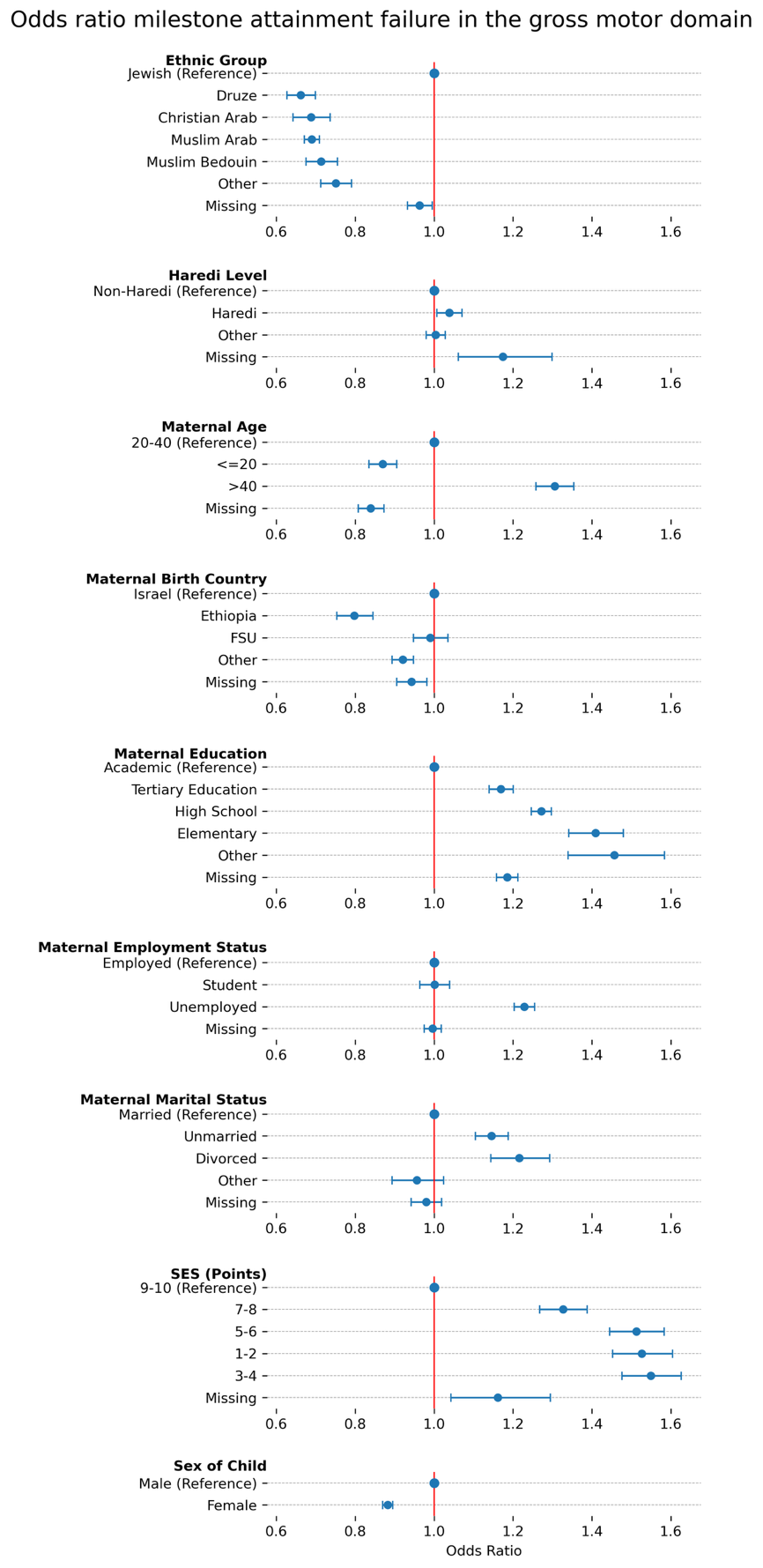


Supplementary Figure 6. Multivariable analysis for failure in the gross motor domain for 2019-2022, where x axis is the odds ratio and y axis is the demographic variables.


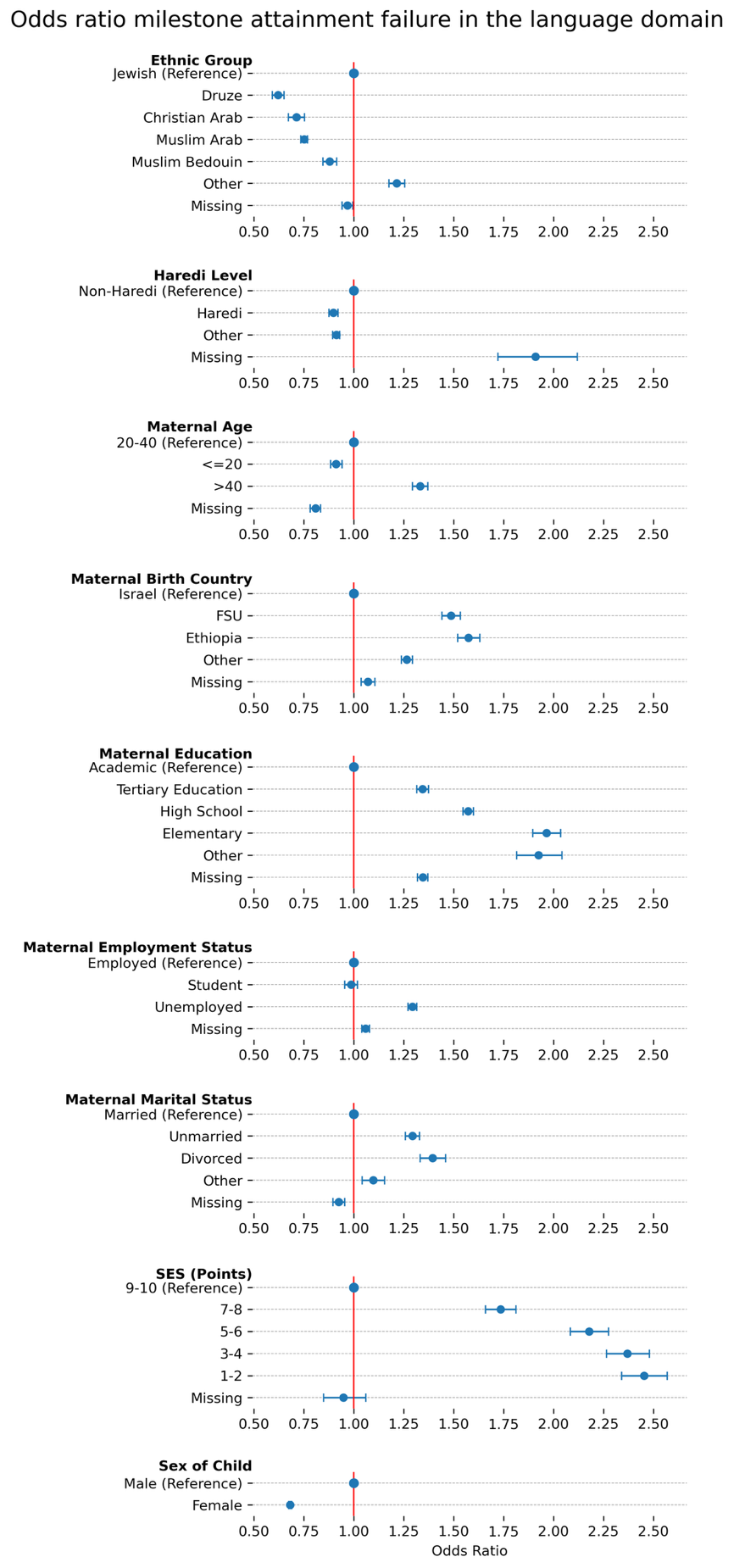


Supplementary Figure 7. Multivariable analysis for failure in the language domain for 2019-2022, where x axis is the odds ratio and y axis is the demographic variables.


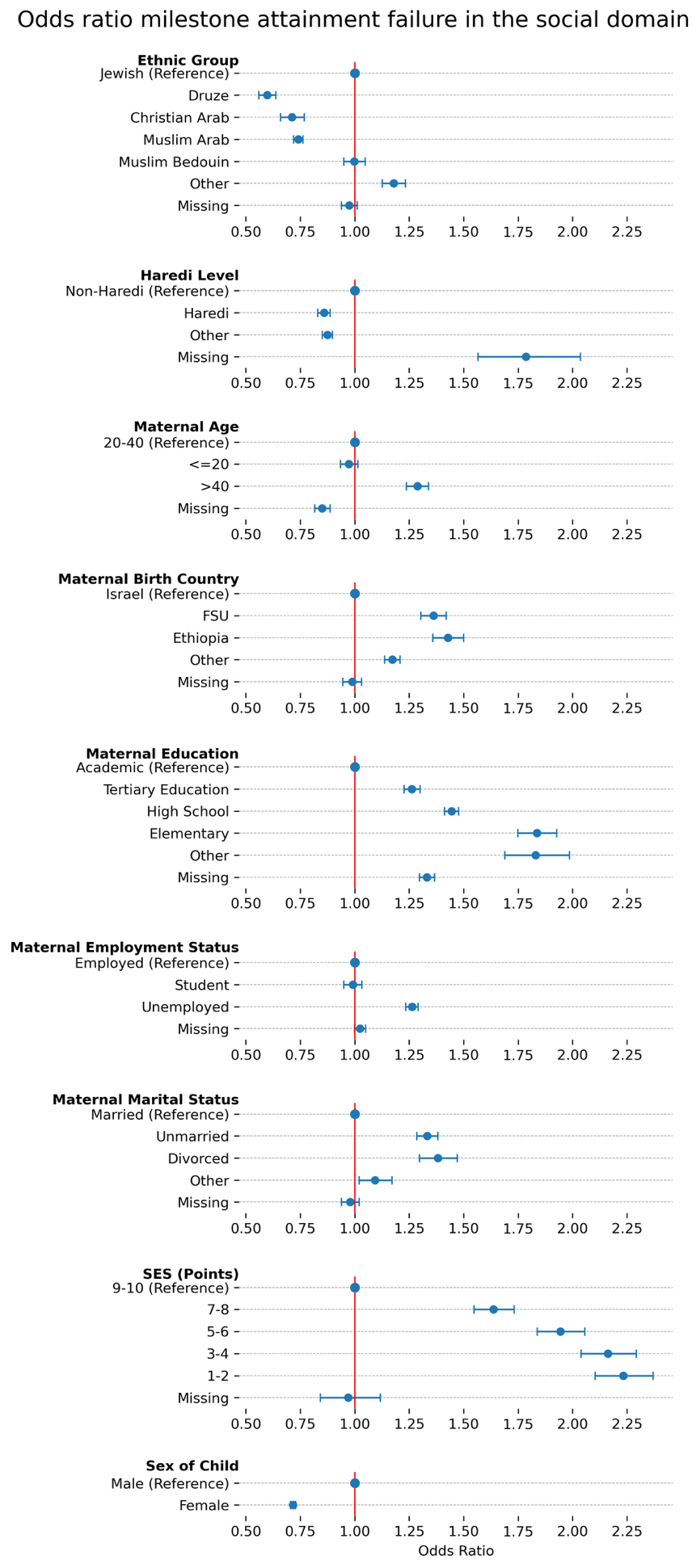


Supplementary Figure 8. Multivariable analysis for failure in the social domain for 2019-2022, where x axis is the odds ratio and y axis is the demographic variables.


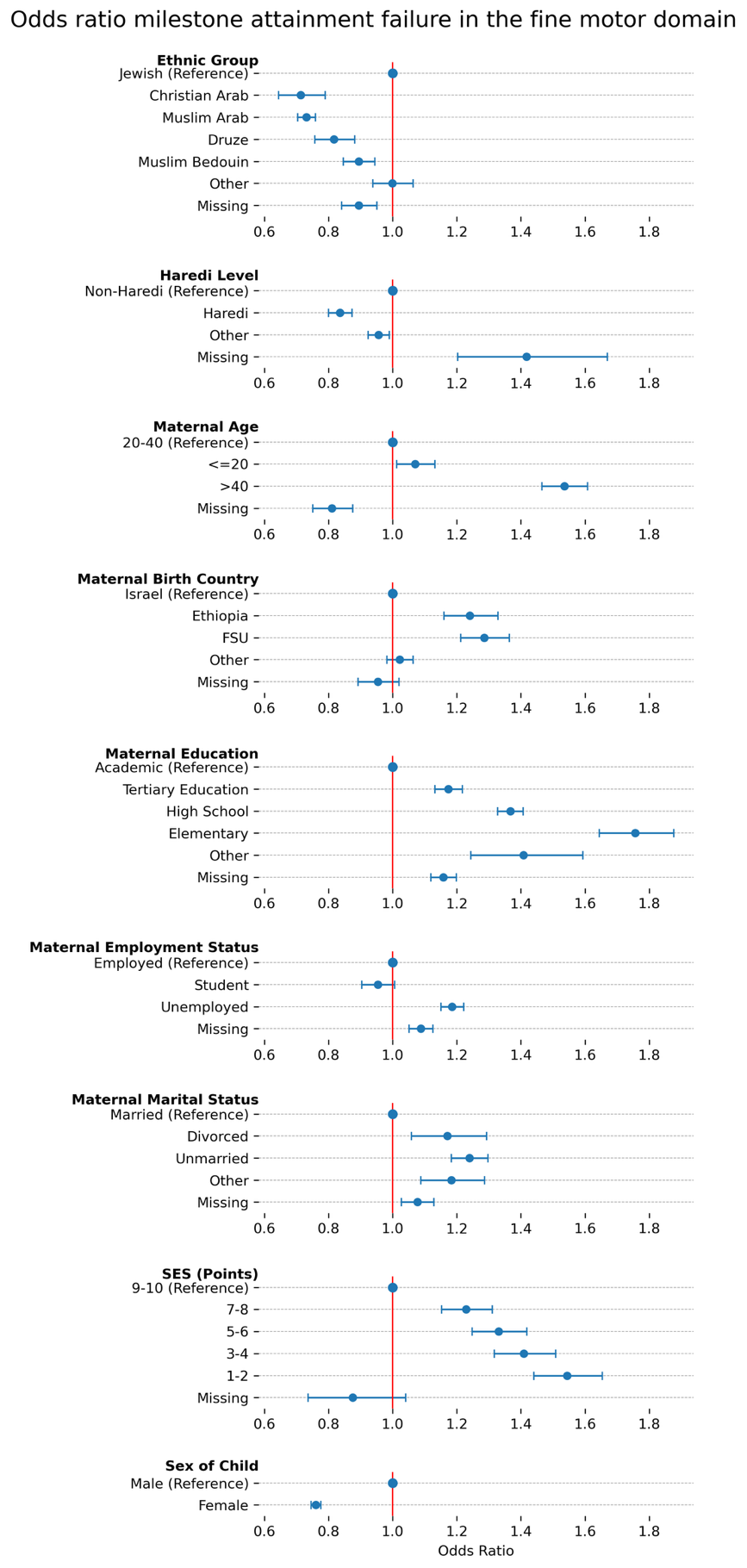


Supplementary Figure 9. Multivariable analysis for failure in the fine motor domain for 2023-2024, where x axis is the odds ratio and y axis is the demographic variables.


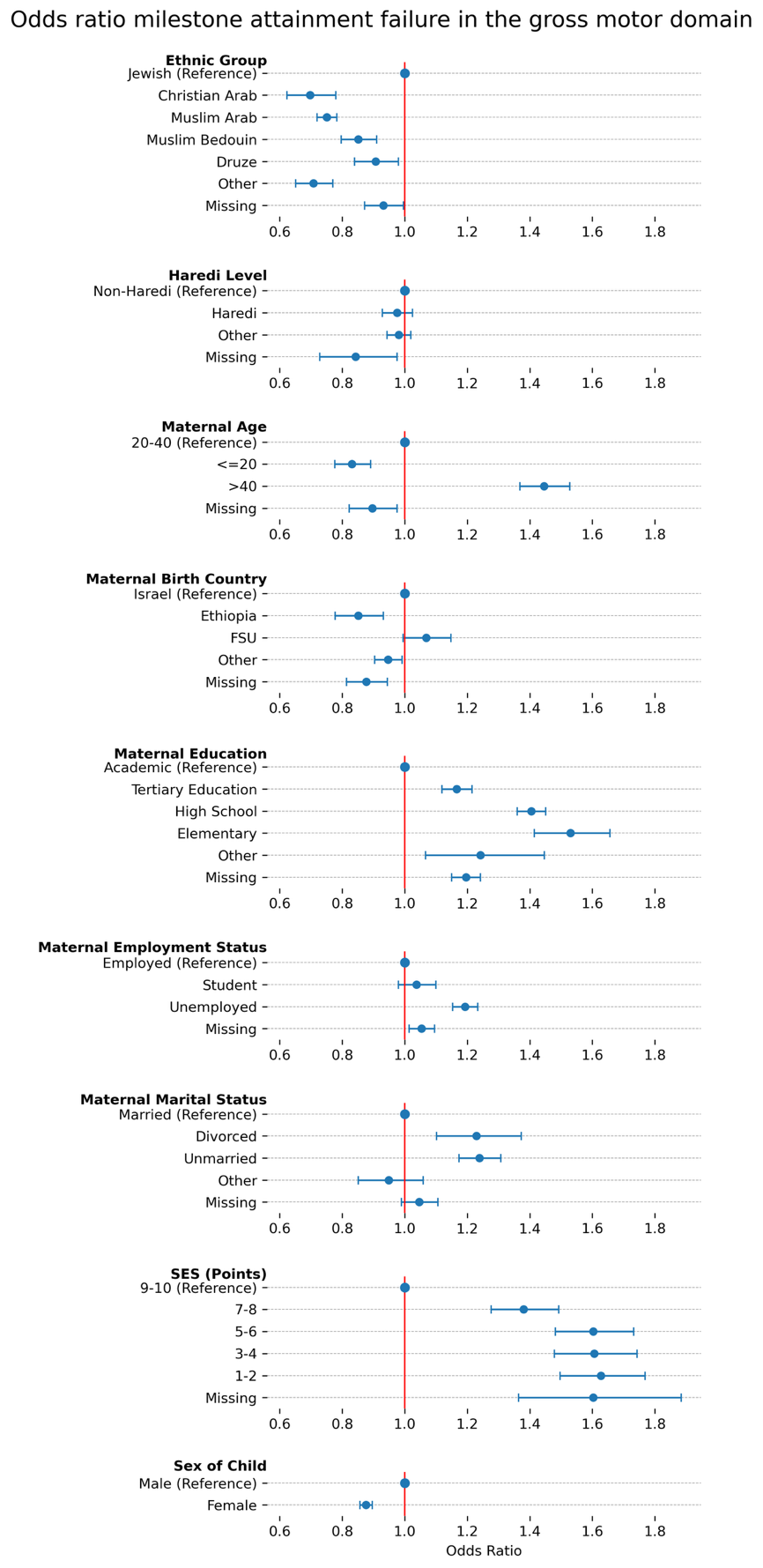


Supplementary Figure 10. Multivariable analysis for failure in the gross motor domain for 2023-2024, where x axis is the odds ratio and y axis is the demographic variables.


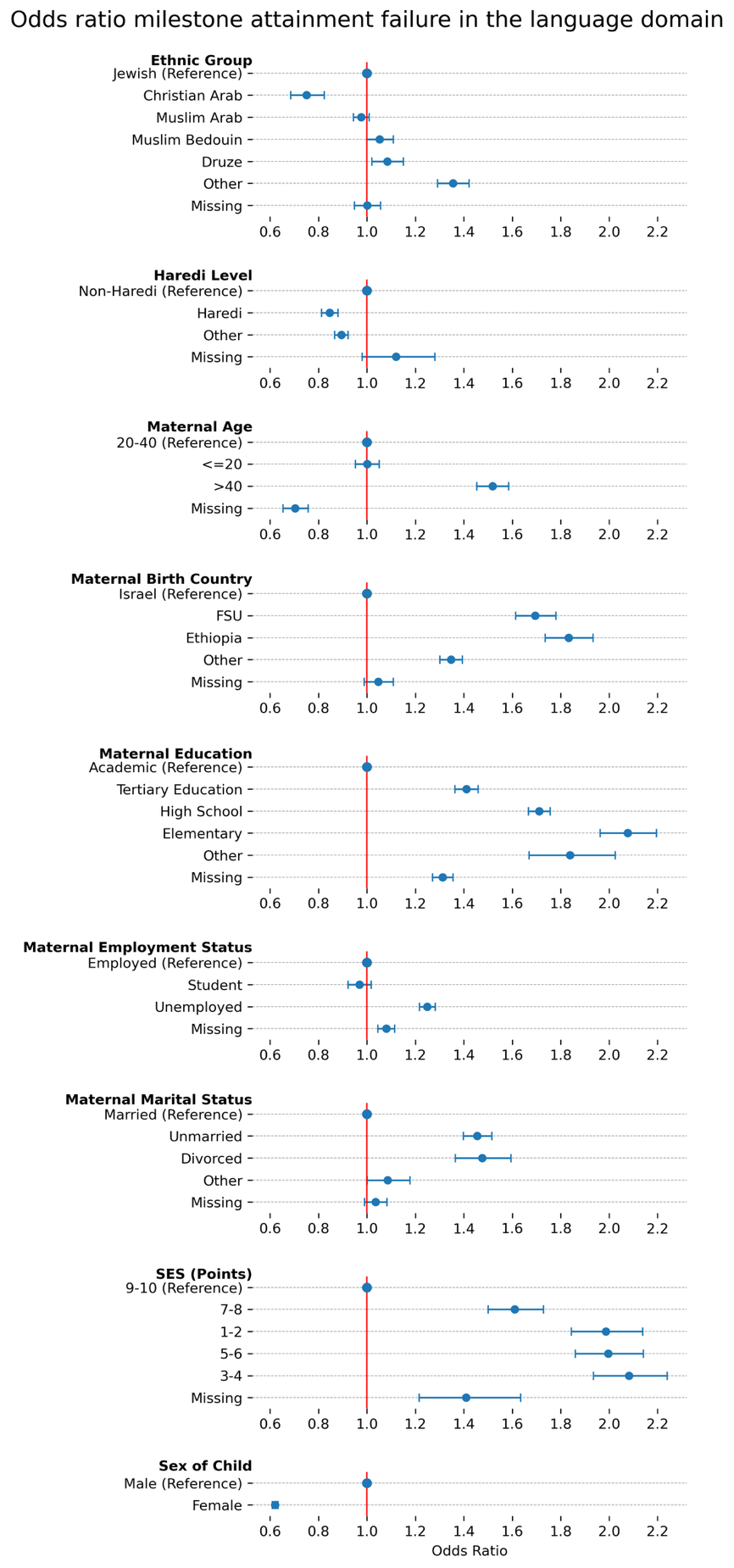


Supplementary Figure 11. Multivariable analysis for failure in the language domain for 2023-2024, where x axis is the odds ratio and y axis is the demographic variables.


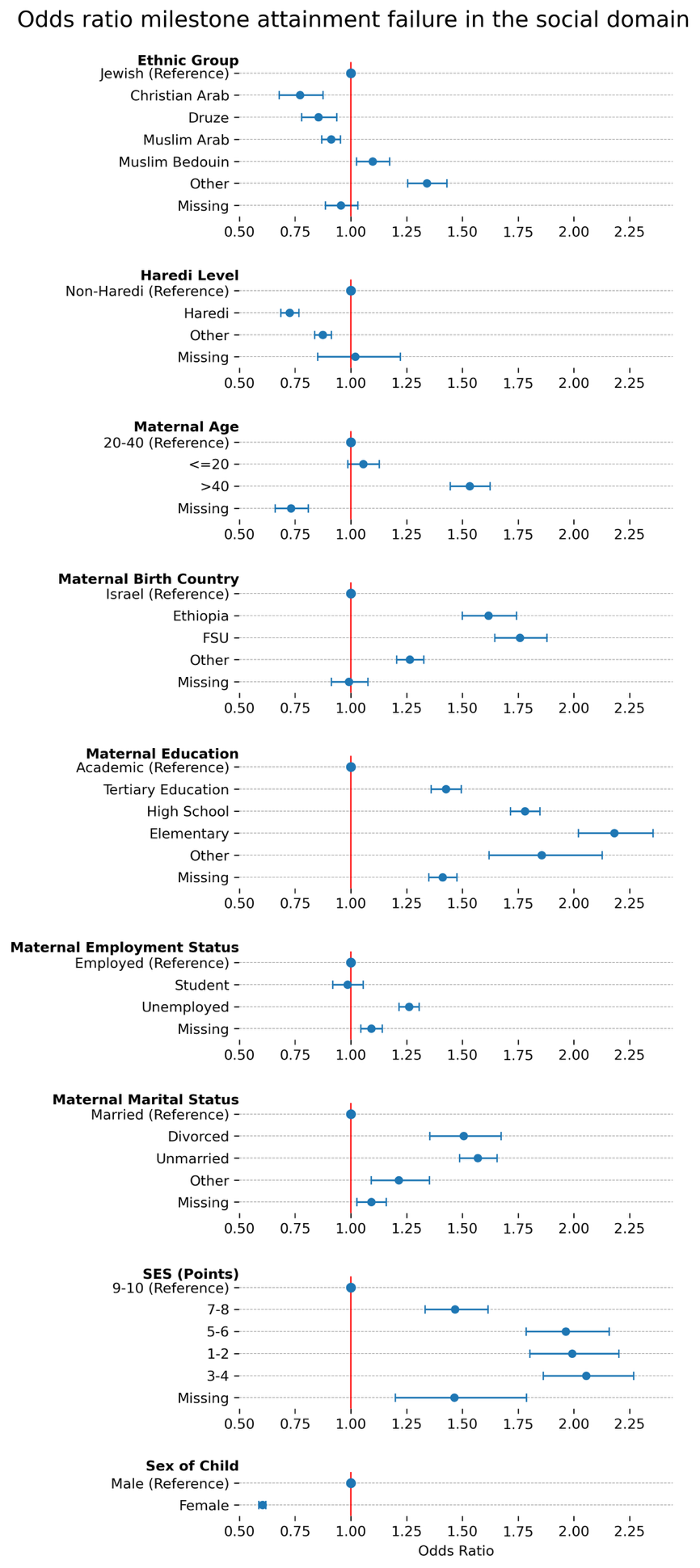


Supplementary Figure 12. Multivariable analysis for failure in the social domain for 2023-2024, where x axis is the odds ratio and y axis is the demographic variables.
